# Pigmented villonodular synovitis of the cervical spine: case report and review of the literature

**DOI:** 10.1259/bjrcr.20150264

**Published:** 2015-10-12

**Authors:** Nicholas A Koontz, Edward P Quigley, Benjamin L Witt, R Kent Sanders, Lubdha M Shah

**Affiliations:** ^1^ Division of Neuroradiology, Department of Radiology, University of Utah Health Science Center, Salt Lake City, UT, USA; ^2^ Division of Anatomic Pathology, Department of Pathology, University of Utah Health Science Center, Salt Lake City, UT, USA; ^3^ Division of Musculoskeletal Imaging, Department of Radiology, University of Utah Health Science Center, Salt Lake City, UT, USA

## Abstract

Pigmented villonodular synovitis (PVNS) is an uncommon, benign, but locally aggressive lesion characterized most commonly by synovial proliferation of the appendicular large joints, but occasionally involving a bursa or the tendon sheath. PVNS of the spine is rare, typically involving the posterior elements. The lytic radiographic appearance and fludeoxyglucose avidity of PVNS may mimic malignant bone lesions, including metastatic disease or myeloma. On *T*_1_ and *T*_2_ weighted, and gradient recalled echo MRI sequences, the low signal intensity may mimic giant cell tumour of the bone, gout or synovial amyloid deposits, thus posing a diagnostic dilemma for the imagers and the treating clinicians. We present a pathologically confirmed case of PVNS of the cervical spine in a 49-year-old female, detailing her imaging work-up, describing histopathological correlation and highlighting the lesion location and involvement of the joint space as useful imaging discriminators for diagnosing PVNS of the cervical spine.

## Summary

Pigmented villonodular synovitis (PVNS) is an uncommon, benign, but locally aggressive lesion characterized by tumefactive synovial proliferation of unknown aetiology.^1^^–^^6^ PVNS typically involves the synovial membrane of large joints, but can also involve the bursae or the tendon sheaths.^2^ PVNS is a rare lesion with a reported incidence of 1.8 million cases per year worldwide.^7^ Spinal involvement is very rare, with most series reporting the cervical spine to be most common site, followed by thoracic and then lumbar spine.^3,6,8^ PVNS classically demonstrates low-to-intermediate signal intensity on all MR pulse sequences, including "blooming artefact" on gradient recalled echo (GRE) sequence. Its lytic radiographic appearance and marked fludeoxyglucose (FDG) avidity can mimic the more aggressive bone lesions, including metastatic disease and myeloma. However, its propensity to involve the posterior elements, facet joints and paraspinal soft tissues makes *location *a useful discriminator for establishing the diagnosis of PVNS on imaging.^6,8^

We report the case of a 49-year-old female with PVNS arising in the atlantoaxial joint with invasion of the C1, C2 and the prevertebral space. We detail her imaging workup, including the CT-guided biopsy technique, describe the histopathological correlation, and highlight the useful imaging discriminators for differentiating PVNS of the cervical spine from other mimicking pathologies. 

## Case report

### Clinical history

A 49-year-old Caucasian female with no pertinent past medical or antecedent trauma history presented to neurosurgery with a 2-year history of progressive neck stiffness and spasm, left-sided jaw pain and left-sided headache. Her symptoms initially started with toothaches in the left jaw, for which a dental examination was normal. She subsequently developed worsening neck spasm and shooting pain extending from the superoposterior left neck into the left occiput, which was typically worse in the morning and aggravated by leaning to the left.

## Examination

Physical examination revealed full range of motion of the neck with forward flexion to 90°, extension to 30° and lateral rotation to 90°. She had moderate tenderness of the paraspinous muscles bilaterally, a negative Lhermitte sign and Spurling test, and no neurological (motor, sensory or deep tendon reflex) deficits. The neck disability index score was rated at 8/50 (16%), in keeping with mild disability.

## Imaging work-up

**Figure 1.**
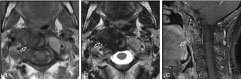
MRI of a 49-year-old female with pigmented villonodular synovitis of the cervical spine. (a) Axial *T*_1_ weighted imaging without intravenous contrast demonstrates a heterogeneously hypointense trans-spatial mass (white open arrows) involving the dens and right lateral mass of C2, the anterior arch and right lateral mass of C1, and the prevertebral space. (b) The mass is markedly hypointense on *T*_2_ weighted imaging. (c) On sagittal contrast-enhanced fat-suppressed *T*_1_ weighted image, the lesion demonstrates faint, heterogeneous enhancement. There is no epidural extension of tumour.

**Figure 2.**
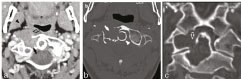
CT imaging of a 49-year-old female with pigmented villonodular synovitis of the cervical spine. (a) A contrast-enhanced CT scan reveals a lytic, heterogeneously enhancing, soft-tissue density mass (black solid arrow) involving the C1 and C2, extending ventrally into the prevertebral component of the perivertebral space (black open arrow). The anterior displacement of the right parapharyngeal space is noteworthy (black arrowhead), as expected from a mass originating in the prevertebral space. (b) Axial bone algorithm NECT demonstrates the lytic lesion (white solid arrow) involving the C1 and C2, including its involvement of the dens (white arrowhead). The sharp zone of transition and thin, sclerotic margins are noteworthy features typically associated with benign, slow-growing lesions. No matrix mineralization is seen. (c) Coronal reconstruction of bone algorithm NECT clearly demonstrates that the lesion is centred about the atlantoaxial joint (white open arrow), a critical diagnostic feature establishing this to be a tumour of synovial origin. NECT, non-contrast-enhanced CT.

**Figure 3.**
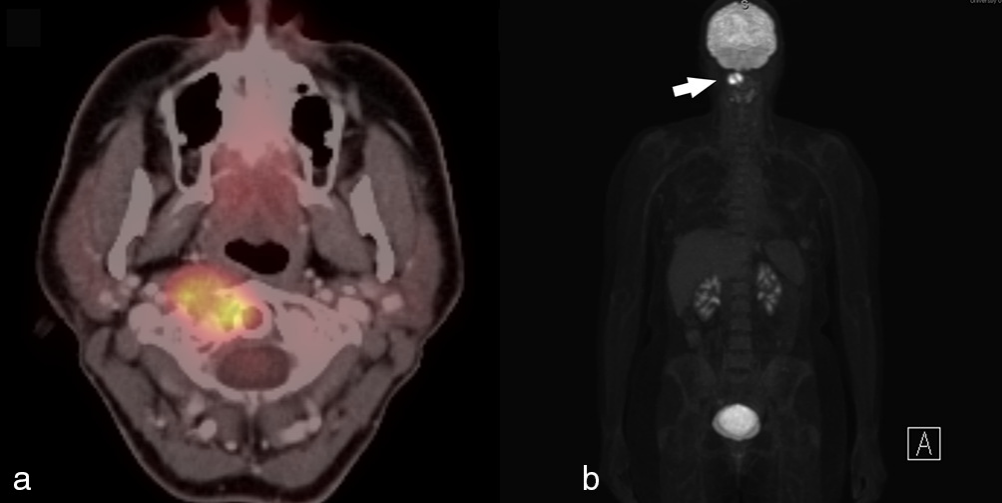
FDG-PET/CT imaging of a 49-year-old female with pigmented villonodular synovitis of the cervical spine. (a) Fused axial FDG-PET/CT image demonstrates the cervical spine lesion to be markedly FDG avid. (b) Coronal FDG-PET image demonstrates the C1/C2 lesion (white solid arrow), but there are no other suspicious foci of FDG uptake. Although seen with malignancy, FDG avidity is a marker of metabolic activity and is a non-specific finding that can also be seen with infectious, inflammatory and metabolic lesions. FDG, fludeoxyglucose; PET, positron emission tomography.

An MRI of the cervical spine was performed, revealing a 3.0 × 2.6 × 2.5 cm (maximal transverse, craniocaudal and anteroposterior diameters) heterogeneously *T*_1_ and markedly *T*_2_ hypointense, faintly enhancing trans-spatial mass involving the dens and right lateral mass of C2, the anterior arch and right lateral mass of C1, and the prevertebral component of the perivertebral space (Figure 1). Contrast-enhanced CT (CECT) revealed a soft-tissue density, lytic lesion with a sharp zone of transition and sclerotic margins (Figure 2). No internal matrix or calcification was present. Critical to establishing the diagnosis, the non-contrast-enhanced CT demonstrated the lesion to be centred about the atlantoaxial joint (Figure 2). Whole-body FDG-positron emission tomography (PET)/CT was performed (Figure 3), revealing the mass to be markedly FDG avid with a maximum standardized uptake value of 24.53. Importantly, no other FDG-avid lesions were detected.

## Differential diagnosis

Based on the findings of the MRI, CECT and FDG-PET/CT, the differential diagnosis remained broad, including neoplastic (metastasis, multiple myeloma/plasmacytoma, giant cell tumour or PVNS), metabolic (brown tumour or amyloid) and inflammatory (gout or calcium pyrophosphate dihydrate crystal deposition).

## CT-guided biopsy technique

**Figure 4.**
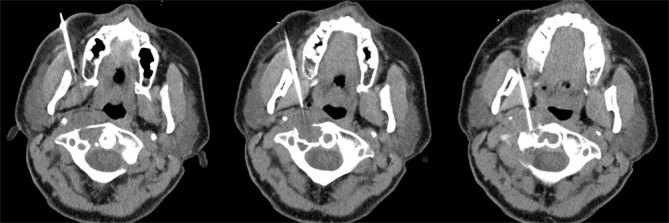
CT-guided trans-facial biopsy images of a 49-year-old female with pigmented villonodular synovitis of the cervical spine. This composite image of three contiguous axial CT images (left = cranial, right = caudal) was obtained during the procedure. This minimally invasive biopsy technique can be performed under moderate sedation in an outpatient setting, avoiding the morbidity and expense of an open biopsy. Pre-procedural consultation with the oncologic surgeons is recommended in order to prevent potential contamination of the planned surgical approach in the event of inadvertent seeding of the biopsy tract with the tumour.

To definitively characterize the lesion, a CT-guided transfacial biopsy was performed with the patient in the supine position and under moderate sedation (Figure 4). Utilizing intermittent CT imaging guidance, a 6.8 cm 17-gauge coaxial needle set was advanced until the tip approximated the C1–C2 mass at the junction of the parapharyngeal and perivertebral spaces. Through the coaxial needle, three fine-needle aspirations were performed with 15 cm 22-gauge Chiba needles (Cook Medical, Bloomington, IN) as well as four core needle biopsies utilizing a 10 cm 18-gauge BioPince biopsy device (Angiotech, Vancouver, BC).

## Pathological findings

**Figure 5.**
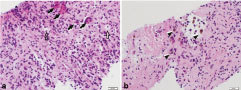
Biopsy specimen of a 49-year-old female with PVNS of the cervical spine. Haematoxylin and eosin (100 × magnification) reveals (a) multinucleated giant cells (black solid arrows), foamy macrophages (black open arrows), and (b) pigmented macrophages (black arrowheads), which are the typical cellular constituents of a PVNS lesion but can also be seen within the solid component of an aneurysmal bone cyst, giant cell tumour of the bone or brown tumour of the bone. Definitive diagnosis of PVNS requires correlation with clinical features and imaging findings, as illustrated in this case. PVNS, pigmented villonodular synovitis.

**Figure 6.**
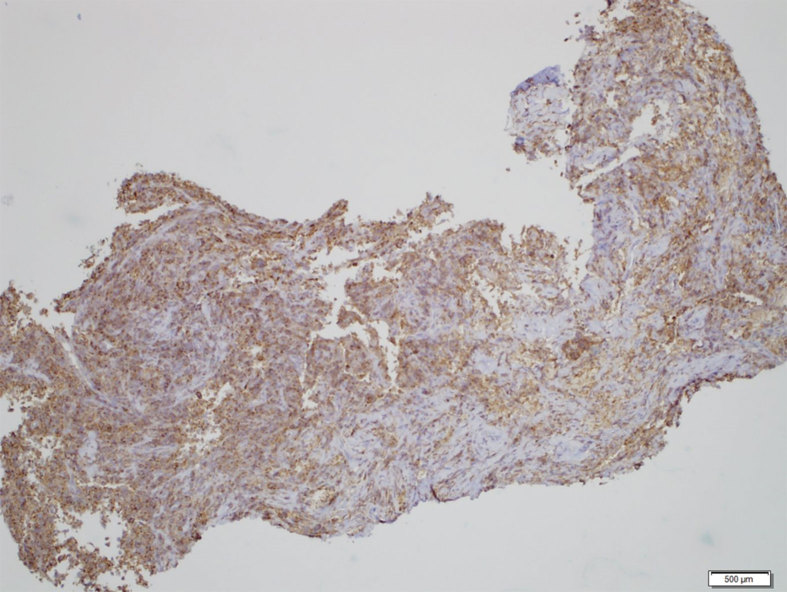
Biopsy specimen of a 49-year-old female with PVNS of the cervical spine. CD68 stain (40 × magnification) demonstrates that the majority of the cells are of histiocytic origin, typical of PVNS. PVNS, pigmented villonodular synovitis.

Tissue cultures (including fungal, acid fast bacilli and anaerobic cultures) were negative for growth and Gram stain was negative for organisms or polymorphonuclear leukocytes. Haematoxylin and eosin preparation slides demonstrated numerous histiocytes (small mononuclear cells showing spindle- to oval-shaped nuclei) with several multinucleated giant cells, foamy macrophages and pigmented macrophages also present (Figure 5). A panel of immunohistochemical stains was performed on paraffin sections from the cell block utilizing appropriately reacting positive and negative controls. The majority of the cells (both the mononuclear and the giant cells) were CD68 positive (Figure 6), indicating a histiocytic differentiation. The stains for CD45, CD1a and S100 were negative.

## Postoperative course

**Figure 7.**
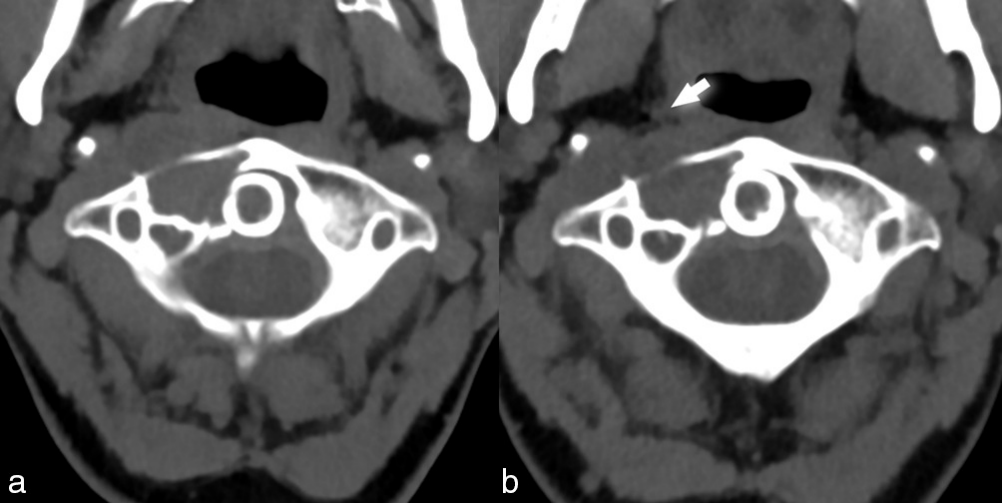
Treatment changes in a 49-year-old female with pigmented villonodular synovitis of the cervical spine. Non-contrast-enhanced CT scan performed pretreatment (a) and 3 months following the initiation of imatinib mesylate therapy (b) reveals subtle reduction in the lesion size, best appreciated in the prevertebral component of the perivertebral space. The reduction in soft-tissue density mass with a more normal contour of the right parapharygeal space (white solid arrow) on post-treatment imaging is noteworthy.

Given the precarious location, risk of operative morbidity and the patient’s desire to preserve range of motion, a decision was made by our institution’s multidisciplinary tumour board to pursue conservative medical management with imatinib mesylate (Gleevec; Novartis, Basel, Switzerland), rather than resection and occipitocervical fusion. Given the paucity of literature regarding long-term treatment of PVNS with imatinib, the treatment plan was to continue this cytostatic medical therapy and make future changes in treatment based upon clinical and imaging findings. Short-interval MR and CT imaging demonstrated a small reduction in lesion size, particularly the prevertebral soft-tissue component (Figure 7). The patient continues to do well clinically, with reduced neck pain from the time of initial presentation.

## Discussion

First described by Jaffe et al^4^ in 1941, PVNS is an uncommon disorder of tumefactive synovial proliferation. It shares common histological features with a related condition called giant cell tumour of the tendon sheath (GCTTS), which demonstrates localized (also known as nodular tenosynovitis), intra-articular and diffuse subtypes.^5^ PVNS is differentiated from GCTTS based upon clinical and imaging manifestations.^5,9^ For practical purposes, PVNS can be thought of as the diffuse intra-articular variant of this spectrum disorder, whereas GCTTS involves the tendon sheath or is found more focally within the joint space. Some authors have suggested that the diffuse form of GCTTS represents an extra-articular extension of PVNS, given its typical location adjacent to the weightbearing joints.^9^ PVNS most commonly involves the knees (reported as high as 80% of cases), and less commonly the hips, ankles, shoulders or elbows. In contradistinction, the most common subtype of GCTTS (localized) typically affects the hand, usually the volar aspect of the first three fingers.^1,5^

Patients with PVNS typically present with non-specific musculoskeletal complaints, including several months of monoarticular joint pain and swelling that may be progressive or wax and wane without specific laboratory or serological findings.^7,9^

## Epidemiology

Among all sites of PVNS involvement, spinal disease is incredibly rare with the literature description limited mainly to small case series by Giannini et al,^3^ Furlong et al^10^ and Motamedi et al,^6^ as well a few case reports. The true incidence of PVNS involving the spine remains unknown. Involvement of the cervical spine is reported most commonly (50–73%), with thoracic (7–25%) and lumbosacral (20–25%) involvement less common.^3,6,10^ It is noteworthy that there is some discrepancy as to the relative frequency of thoracic and lumbosacral PVNS, which may be owing to the small population sizes of the published series. Additionally, it remains to be seen whether cervical PVNS is truly more common or if the statistical predominance is a product of selection bias owing to these being more clinically symptomatic lesions.^6^

While there is some variability between the reported case series, no sex predilection for PVNS in general has been established.^1,6,7^ However, a slight female predominance of PVNS of the spine has been reported (64%).^6^ PVNS has been reported across a wide age range (11–84 years), but it is most commonly seen in the third and fourth decades of life.^1,5,7^

## Location

Based upon data from the three aforementioned series, PVNS of the spine almost invariably (> 90%) involves the posterior elements.^3,6,10^ In the largest published series (15 patients with pathologically proven PVNS of the spine), Motamedi et al^6^ reported spine PVNS to almost always involve the facet joint (93%) and the paraspinal soft tissues (93%), as well as frequently involving the neural foramina (73%), pedicles (67%) and lamina (67%); in their series, PVNS uncommonly involved the vertebral body (27%) or the spinous process (7%). Similarly, our case showed involvement of the neural arch and prevertebral soft tissue extension. Extension into the epidural space is frequent, reported in up to 70% of cases in a small series that evaluated 10 patients for epidural involvement.^3^ Additionally, invasion of the foramen transversarium and involvement of the vertebral artery was reported in multiple patients by Motamedi et al,^6^ although the small number of patients in this series limits the scope of applicability of this finding.

## Imaging

Spinal PVNS lesions are best evaluated with cross-sectional imaging, with complimentary information obtained from CT scan and MRI. A CT scan is often the first-line imaging modality for these patients and is useful for evaluating the margins, matrix, zone of transition and lesion centre. As reported by Motamedi et al,^6^ CT scan findings of PVNS include a lytic soft-tissue mass that is isodense to skeletal muscle (100%), has a defined margin (83%) and typical homogeneous attenuation (67%).^6^ Internal calcification is not expected and its presence should direct one to consider alternative diagnoses.^2,6^ An MRI is helpful in characterizing the soft-tissue component. Spinal PVNS, as in our case, demonstrates a defined margin (100%) with intermediate *T*_1_ (100%) and intermediate (67%) or low (33%) *T*_2_ signal intensity.^6^ Heterogeneity of signal intensity is common. As with PVNS of the large joints, susceptibility artefact from haemosiderin on *T*_2_* GRE sequences is suggestive of the diagnosis, but may be variably seen owing to the relative amounts of lipid, fibrous tissue, cystic content, cellular constituents and haemosiderin within individual lesions.^8^ Diffuse and moderate-to-marked contrast enhancement is typical.^6,8^ Finally, one must keep in mind that PVNS may demonstrate marked FDG avidity, as in our case, mimicking metastases and aggressive, malignant bone tumours.^11,12^

## Pathology

As aforementioned, PVNS and GCTTS share common histopathological features of histiocytes, giant cells, pigmented (haemosiderin laden) and foamy (lipid laden) macrophages, with the diagnosis dependent upon specific clinical features.^1,3,5,7^ Further complicating the pathological diagnosis is the histopathological similarity of PVNS to other diagnoses, including giant cell tumour of the bone, aneurysmal bone cyst (ABC), and brown tumour of the bone. This requires the interpreting pathologist to utilize multiple tools, including analysis of the cellular constituents, immunohistochemistry, radiological findings and clinical features to reach the correct final diagnosis. In this case, the predominant histiocytic component with scattered giant cells and pigmented macrophages is more typical of PVNS, whereas giant cell tumour of the bone typically shows a repetitive, sheet-like population of mononuclear cells with similar-appearing nuclei and repetitive, uniformly distributed osteoclast-like giant cells. The imaging findings of a soft-tissue density, lytic lesion centred about a synovial joint space clinched the diagnosis of PVNS for the pathologist. In our case, the lesion lacked the typical imaging features (i.e. fluid–fluid levels) of ABC, the subchondral/subcortical location of giant cell tumour of the bone and the typical clinical profile (i.e. hyperparathyroidism) of brown tumour.

## Treatment

Gross-total surgical resection is the recommended first-line therapy for PVNS of the spine.^3^ As with appendicular disease, local recurrence of PVNS following surgery is not uncommon, reported in approximately 20% of cases.^3,13^ In complicated cases precluding gross-total resection, subtotal resection with close clinical follow-up has traditionally been advised.^3,14^ More recently, imatinib mesylate has been identified as a potential therapeutic agent for the treatment of unresectable, incompletely resectable or recurrent PVNS.^15,16^ A tyrosine kinase inhibitor, imatinib inhibits colony-stimulating factor 1 (CSF1), which is overexpressed in 30–60% of patients with PVNS, thus inhibiting CSF1-mediated chemotaxis and resulting in symptomatic improvement in a majority of patients (73%) in a small, multicentre trial.^16^ However, there remains a paucity of literature providing long-term guidelines for the use of imatinib as treatment of PVNS. Additionally, the use of post-operative external-beam radiation following complete or cytoreductive surgery has been reported as an effective means of preventing recurrent PVNS; despite the potential risks of impaired wound healing, radiation-induced neurological damage and the small risk of radiation-induced neoplasia inherent with all radiation therapy, modern treatment planning and typical doses between 30–50 Gy allow for improved control rates of PVNS relative to surgical management alone and confer a minimal risk of neurological injury.^3,14,17–19^

## Conclusions

PVNS of the spine is a rare, benign, but locally aggressive lesion that can mimic malignancy, metabolic or inflammatory disease on CT scan, MRI and FDG-PET, resulting in a diagnostic dilemma for imagers, clinicians and pathologists. As reported in this instructive case of PVNS of the cervical spine, location of the lytic soft-tissue lesion about the joint space is a critical diagnostic discriminator.

## Learning points

1. PVNS of the spine is rare, but when it does occur in the cervical spine, it typically involves the posterior elements.
2. PVNS classically has a lytic radiographic appearance and demonstrates low-to-intermediate signal intensity on all MR pulse sequences.
3. The intra-articular location of PVNS can be used to differentiate it from GCTTS, which involves the tendon sheath or is found more focally within the joint space.
